# Respiratory virus type to guide predictive enrichment approaches in the management of the first episode of bronchiolitis: A systematic review

**DOI:** 10.3389/fimmu.2022.1017325

**Published:** 2022-10-27

**Authors:** Dominika Ambrożej, Heidi Makrinioti, Abigail Whitehouse, Nikolas Papadopoulos, Marek Ruszczyński, Aleksander Adamiec, Jose A. Castro-Rodriguez, Khalid Alansari, Tuomas Jartti, Wojciech Feleszko

**Affiliations:** ^1^ Department of Pediatric Pneumonology and Allergy, Medical University of Warsaw, Warsaw, Poland; ^2^ Doctoral School, Medical University of Warsaw, Warsaw, Poland; ^3^ Harvard T.H. Chan School of Public Health, Boston, MA, United States; ^4^ Centre for Genomics and Child Health, Queen Mary University of London, London, United Kingdom; ^5^ Division of Infection, Immunity and Respiratory Medicine, University of Manchester, Manchester, United Kingdom; ^6^ Allergy Department, 2nd Pediatric Clinic, University of Athens, Athens, Greece; ^7^ Department of Pediatrics, Medical University of Warsaw, Warsaw, Poland; ^8^ Department of Pediatric Pulmonology and Cardiology, School of Medicine, Pontificia Universidad Católica de Chile, Santiago, Chile; ^9^ Department of Pediatric Emergency Medicine, Sidra Medicine, Doha, Qatar; ^10^ Clinical Pediatrics, Qatar University College of Medicine, Doha, Qatar; ^11^ Clinical Pediatrics, Weill Cornell Medical College- Qatar, Doha, Qatar; ^12^ Department of Pediatrics, Turku University Hospital and University of Turku, Turku, Finland; ^13^ PEDEGO Research Unit, Medical Research Center, University of Oulu, Oulu, Finland; ^14^ Department of Pediatrics and Adolescent Medicine, Oulu University Hospital, Oulu, Finland

**Keywords:** precision medicine, infant, bronchiolitis, viruses, corticosteroids, asthma, rhinovirus

## Abstract

It has become clear that severe bronchiolitis is a heterogeneous disease; even so, current bronchiolitis management guidelines rely on the one-size-fits-all approach regarding achieving both short-term and chronic outcomes. It has been speculated that the use of molecular markers could guide more effective pharmacological management and achieve the prevention of chronic respiratory sequelae. Existing data suggest that asthma-like treatment (systemic corticosteroids and beta2-agonists) in infants with rhinovirus-induced bronchiolitis is associated with improved short-term and chronic outcomes, but robust data is still lacking. We performed a systematic search of PubMed, Embase, Web of Science, and the Cochrane’s Library to identify eligible randomized controlled trials to determine the efficacy of a personalized, virus-dependent application of systemic corticosteroids in children with severe bronchiolitis. Twelve studies with heterogeneous methodology were included. The analysis of the available results comparing the respiratory syncytial virus (RSV)-positive and RSV-negative children did not reveal significant differences in the associatons between systemic corticosteroid use in acute episode and duration of hospitalization (short-term outcome). However, this systematic review identified a trend of the positive association between the use of systematic corticosteroids and duration of hospitalization in RSV-negative infants hospitalized with the first episode of bronchiolitis (two studies). This evidence is not conclusive. Taken together, we suggest the design for future studies to assess the respiratory virus type in guiding predictive enrichment approaches in infants presenting with the first episode of bronchiolitis.

**Systematic review registration:**

https://www.crd.york.ac.uk/prospero/, identifier CRD42020173686

## Introduction

It is believed that the first severe episode of wheezing, or severe bronchiolitis, may be the first sign of developing asthma; therefore, immunological methods for its early detection are being sought to implement efficient management early (1, 2). In addition, mechanistic studies have shown that viral respiratory infections can contribute to type 2 inflammation (3, 4). The restriction to access to airway samples in infants has complicated the investigation of host immune responses to respiratory viruses, limiting our understanding of these processes and, thus, impacting the appropriate choice of therapeutic strategies that can benefit children presenting with the first episode of bronchiolitis.

The current conservative bronchiolitis management relies on the one-size-fits-all approach regardless of short-term and chronic outcomes. However, the common clinical practice based on the use of systemic corticosteroids in bronchiolitis has not been supported by any systematic review. Thus, systemic corticosteroids are not recommended as the first-line treatment in any international guidelines for managing bronchiolitis and mainstream bronchiolitis care is solely supportive (5–9).

On the other hand, increasing evidence demonstrates that bronchiolitis is a heterogeneous disease and that a viral trigger may be one of the key exposures and part of the underlying pathobiology, very important in identifying endotypes (2, 10, 11). Although the clinical features of bronchiolitis attributed to different viruses are usually indistinguishable, the recently recognized bronchiolitis profiles are associated with various risks for recurrent wheeze and asthma, some differences in disease severity, and, potentially, different therapeutic responses to systemic corticosteroids (10–13). For example, it has been shown that human rhinovirus (HRV)-associated bronchiolitis can result in shorter hospitalization times than bronchiolitis caused by the respiratory syncytial virus (RSV); however, the evidence around associations between virus type and severity is still unclear (14, 15). While most children hospitalized due to bronchiolitis have an uneventful course, it should be remembered that approximately 2-6% require admission to pediatric intensive care units and invasive mechanical ventilation, or even occasionally be fatal (16–18).

Likewise, current studies evaluated the efficacy of systemic corticosteroid therapy in severe bronchiolitis (19–21). In evaluating these studies, one should remember that the sample size was not homogeneous. Therefore, other confounding factors, such as respiratory virus type or peripheral blood eosinophilia or parental history of asthma/allergy, were not evaluated. Furthermore, the effectiveness of the systemic corticosteroids may not depend on the virus type per se but potentially on the eosinophilic airway inflammation, which is often present in atopic children and/or accompanied by a HRV-induced respiratory infection (22, 23).

Recognition of the variability of bronchiolitis, coupled with the failure to identify effective therapies, has provided incentives to establish a precision medicine approach in bronchiolitis management (24). Precision medicine refers to the customization of diagnostic and therapeutic processes based on the unique features of an individual patient (25, 26), and this concept is called the “concept of enrichment” (27). Prognostic enrichment reflects the selection of patients more prone to a disease-related event, such as mortality. Meanwhile, predictive enrichment refers to selecting patients more likely to respond to therapy based on the biological mechanism.

Currently, no clear evidence suggests using respiratory virus testing as a guide for systemic corticosteroids in the first episode of severe bronchiolitis. Therefore, we aimed to systematically revise the literature on whether the respiratory virus type can guide predictive enrichment approaches in the first episode of bronchiolitis.

## Methods

### Search strategy

The study followed the Preferred Reporting Items for Systematic Reviews and Meta-Analyses (PRISMA) guidelines (28) and was registered in the National Institute for Health Research’s PROSPERO (CRD42020173686).

Four bibliographic databases were searched (PubMed, Web of Science, Embase, and Cochrane’s Library) from inception to September 23, 2019. Additionally, the updated search was conducted closer to submission (January 04, 2022). The search term (*steroid* OR *predniso* OR dexamethasone) AND (wheez* OR bronchiolitis) was followed using a Boolean methodology. All extracted citations were imported into EndNote^®^ reference manager (Version X8, Clarivate Analytics, 2016). After removing the duplicates, two reviewers (DA and HM) working independently screened the retrieved titles and abstracts.

Subsequently, all potentially relevant publications were assessed in full text. At each stage, uncertainty about the eligibility of studies for the review was resolved through discussion and through obtaining consensus by other reviewers (AW, NP, WF, TJ), if necessary.

### Eligibility criteria

Main eligibility criteria included publication in English language, randomized controlled trial design (RCTs), age group under two years old, the clinical presentation with the first episode of bronchiolitis or wheezing episode at the hospital setting (emergency department (ED) and/or ward), intervention with the administration of systemic corticosteroids, that assessed viral etiology of the illness.

Exclusion criteria included duplicate publications, non-human studies, RCTs that did not involve management at the ED or admission to the paediatric ward, RCTs including children presenting with confirmed one viral agent (e.g., only RSV-positive cases), RCTs including children who received inhaled corticosteroids as the trial intervention, study protocols, editorials or review papers, and conference abstracts.

Actions were taken to contact corresponding authors when additional clarification and further data were required.

Our primary outcomes were viral-dependent short-term outcomes, such as change in the baseline clinical severity scores (i.e., the Respiratory Distress Assessment Instrument, RDAI), need for oxygen therapy, hospitalization rate in the ED studies, and length of hospitalization in the inpatient studies. The secondary outcomes of our review included viral-dependent long-term outcomes (two months or longer after the study enrollment) such as re-admission to medical center due to respiratory symptoms or initiation of regular controller medication for asthma symptoms.

### Study extraction and synthesis

Data were extracted from each included study for the following parameters: (a) study origin, (b) participant details, (c) diagnostic intervention(s) and the type of systemic corticosteroids used, (d) administered control, (e) short-term and long-term outcomes, (f) details on results assessed per viral etiology, (g) overall risk of bias assessment, and (h) potential confounding factor(s).

Given the small number of studies utilizing similar methodology and describing virus detection data, meta‐analysis was not possible, and consequently, a narrative synthesis method was used.

### Risk of bias assessment

Two independent reviewers (DA, HM) assessed the risk of bias in each of the included studies without being blinded to the authors or journal. A revised Cochrane risk-of-bias tool for randomized trials was used (RoB 2) (29). Encountered discrepancies were resolved through a discussion of all the reviewers. The RoB 2 tool is structured into five bias domains, which enables to judge the randomization process, deviations from intended interventions, any missing outcome data, measurement of the outcome, selection of the reported result.

The judgments for each domain were to choose between “low risk of bias”, “some concerns”, or “high risk of bias”. In conclusion, the overall bias was determined by reflecting the individual marks. The graphical summary of the risk of bias assessment was performed using the robvis online tool (30).

## Results

### Description of the studies

**Figure 1:**
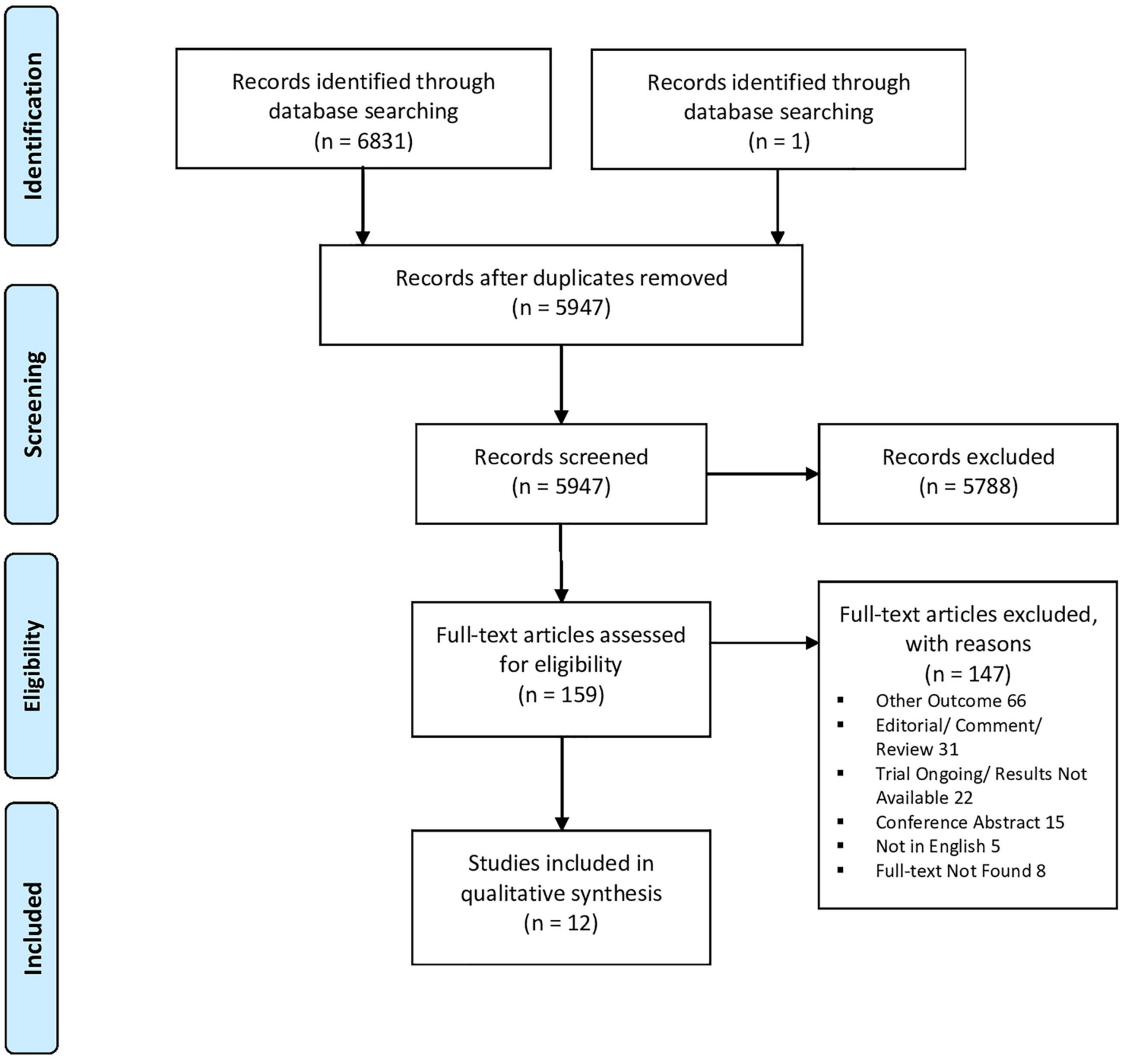
Prisma flow diagram of the study.

Following the systematic search, 6831 records were obtained, including 5947 unique citations. The PRISMA flow chart is shown in **Figure 1**. The screening of titles and abstracts excluded 5788 articles, rendering 159 to full-text assessment. Finally, twelve studies fulfilling the inclusion criteria for systemic application of corticosteroids in the first episode (corticosteroid-naïve) of bronchiolitis and details on the performed viral detection were included (n=1931, see **Table 1**) (31–41, 43). The included studies were conducted in different parts of the world – three studies in Europe (Ireland, Finland) (33, 38, 43), one study in South America (Paraguay) (41), six in North America (USA, Canada) (31, 34, 36, 37, 39, 40), and two in Asia (32, 35). Two of the studies were multicenter (39, 40).

**Table 1:** Characteristics of the included studies in the magnitude of short-term outcome assessment.

Study ID, country	Study design and analysis method	Population and randomized sample size (n), attrition (%)	RSV-positive vs. RSV-negative	Intervention/Comparison groups	Short-term outcome(s) measured	Outcomes assessed as per viral etiology
Connolly et al. (1969) (31), Ireland	RCT, PP	Children up to 24 months of age hospitalized with bronchiolitis (n=100)	RSV-positive (n=78) RSV-negative (n=15)	Oral prednisolone (6 days)/Placebo	Duration of hospitalization Duration of illness from symptoms onset	No significant difference in the duration of illness between compared the RSV-positive and RSV-negative groups
Roosevelt et al. (1996) (32), USA	RCT, PP	Infants up to 12 months of age hospitalized with the first wheezing episode (n=122)	RSV- positive (n=79) RSV-negative (n=39)	Intramuscular DM (max. 3 days)/Placebo	Time to resolution of respiratory symptoms Duration of oxygen therapy	No significant differences in the time to resolution of symptoms (HR (95% CI): 1.2 (0.8–1.9), 1.1 (0.5–2.1), respectively), and the duration of oxygen therapy (HR (95% CI): 0.9 (0.6–1.5), 0.7 (0.3–1.4), respectively) compared the RSV-positive and RSV-negative groups
Klassen et al. (1997) (33), Canada	RCT, PP	Infants up to 15 months of age hospitalized with first wheezing episode (n=72)	RSV-positive (n=58) RSV-negative (n=9)	Oral DM (min. 3 days)/Placebo	Change in the RDAI score at 24 hours after enrollment Time until ready for discharge Medical visits after a 1-week follow-up Medication at discharge	NA
Berger et al. (1998) (34), Israel	RCT, PP	Children 2-18 months of age with the first episode of bronchiolitis (n=42)	RSV-positive (n=19) RSV-negative (n=19)	Oral prednisolone (3 days)/Placebo	Clinical evaluation after 3 days of enrollment Clinical evaluation after 7 days of enrollment	NA
Goebel et al. (2000) (35), USA	RCT, PP	Children <24 months of age with the first wheezing LRTI (n=51)	RSV-positive (n=26) RSV-negative (n=22)	Oral prednisolone (5 days)/Placebo	Bronchiolitis clinical severity score at study days 0, 2, 3, 6 according to the enrollment Hospitalization rate	No significant difference in the clinical improvement between the study’s measurement timepoints compared RSV-positive and RSV-negative
Schuh et al. (2002) (36), Canada	RCT, ITT	Children <24 months of age with the first wheezing bronchiolitis (n=71)	RSV-positive (n=30) RSV-negative (n=28)	Single dose of oral DM/Placebo	RACS after the 4-hour observation period Differences in hospitalization rates after the 4-hour observational period Changes in transcutaneous oxygen saturation Difference in RACS from baseline to day 7	No significant difference in the hospitalization rate between the RSV-positive and RSV-negative groups
Jartti et al. (2006) (37), Finland *the Vinku study*	RCT, PP	Children up to 24 months of age, with the first wheezing bronchiolitis* (n=113)	RSV-positive (n=48) RSV-negative (n=65)	Oral prednisolone (3 days)/Placebo	Time until ready for discharge Oxygen saturation, wheeze, and cough during 2-week follow-up Blood eosinophil counts at dis- charge and at 2-week follow-up	The mean LOS shorter in the RSV-negative children receiving intervention vs. placebo (mean ± SD: 19,7 ± 31,5 vs. 40,0 ± 31,3 hours, P=.002) No significant difference in the mean LOS in the RSV-positive children between the intervention and placebo groups (mean ± SD: 57,2 ± 39,9 vs. 47,6 ± 38,7 hours, P>.05)
Corneli et al. (2007) (38), USA	RCT, ITT (hospital admissions) and PP (RACS)	Infants 2-12 months of age with the first episode of bronchiolitis (n= 600)	RSV-positive (n=166) RSV-negative (n=103)	Single dose of oral DM/Placebo	Hospitalization rate after a 4-hour observation period RACS after a 4-hour observation period	No significant difference in the hospitalization rate and the RACS after 4 hours compared the RSV-positive and RSV-negative groups
Plint et al. (2009) (39), Canada	RCT, ITT	Infants 6 weeks – 12 months of age with the first episode of bronchiolitis (n=401)	RSV-positive (n=263) RSV-negative (n=138)	Two of four study treatment groups: Oral DM plus nebulized placebo (6 days)/Oral and inhaled placebo	Hospitalization rate after a 7-day follow-up Change in heart and respiratory rate, RDAI score, and oxygen saturation from baseline to 30, 60, 120, and 240 minutes after the intervention Time until ready to discharge	No significant difference in the hospitalization rate between the RSV-positive and RSV-negative groups
Mesquita et al. (2009) (40), Paraguay	RCT, PP	Children 2-24 months of age with the first wheezing bronchiolitis (n=80)	RSV-positive (n=36) RSV-negative (n=29)	Single dose of oral DM/Placebo	Change in the RDAI score at the 4-hour observation period Hospitalization rate	No significant difference in the hospitalization rate and the RDAI score at 4-hour observation compared the RSV-positive and RSV-negative groups
Alansari et al. (2013) (41), Qatar	RCT, PP	Infants up to 18 months of age hospitalized the first episode of bronchiolitis with a history of eczema or with a family history of asthma (n=200)	RSV-positive (n=77) RSV-negative (n=123)	Oral DM (5 days)/Placebo	Early discharge within a 46-hour period Medical re-presentations within a 1-week follow-up	Readiness for discharge at 48 hours higher in the RSV-negative vs. the RSV-positive group (OR 0.28, CI95% 0.09-0.92, P=.03)
Jartti et al. (2015) (42), Finland *the Vinku2 study*	RCT, PP	Children 3-23 months of age with the first wheezing episode due to a HRV confirmed infection, other viral co-infections included (n=79)	RSV-positive (n=10) RSV-negative (n=69)	Oral prednisolone (for 3 days)/Placebo	Post-episode respiratory symptoms a 2-week follow-up Use of bronchodilators a 2-week follow-up Medical re-presentations within a 2-week follow-up	No significant differences in any of the assessed outcomes compared the RSV-positive and RSV-negative groups The prednisolone HRV-positive group had less cough, rhinitis, noisy breathing, severe breathing difficulties, and nocturnal respiratory symptoms within 2 weeks (all P<.05)

PP, Per Protocol; ITT, Intention to Treat; RSV, Respiratory Syncytial Virus; HRV, Rhinovirus; NA, Not Available; DM, Dexamethasone; Respiratory Assessment Change Score, RACS; Respiratory Distress Assessment Instrument, RDAI; LOS, Length of hospital Stay; ED, Emergency Department.

*Detailed data on subgroup analysis of children under the age of 2 years and with the first wheezing episode received from the study investigators.

**Figure 2:**
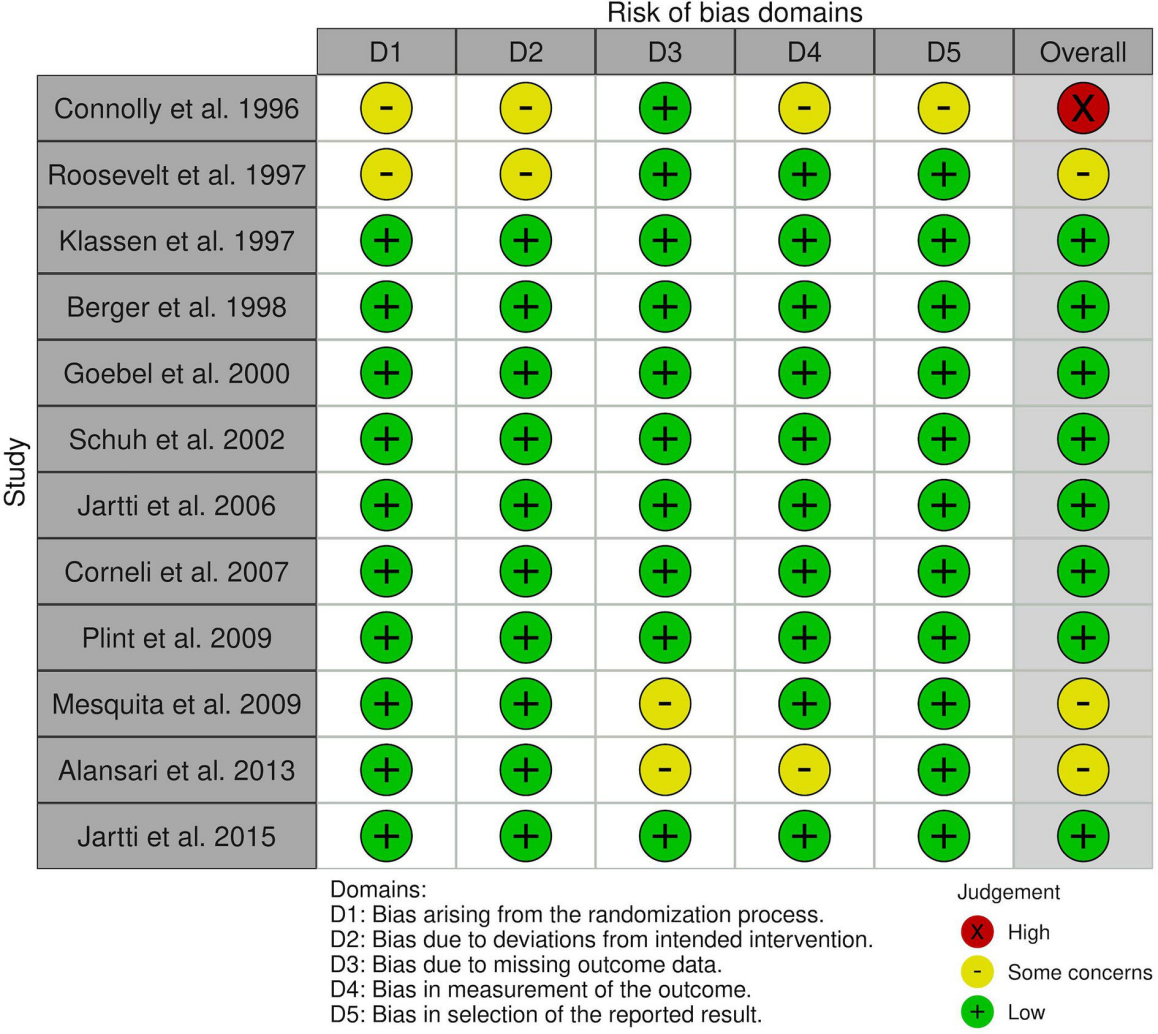
Summary of risk of bias assessment of the eligible studies.

Among the included studies, the overall risk of bias was considered low (33–38) in six, or only with some concerns in three of them (31, 32, 41) (**Figure 2**). However, one of the studies were judged as with a high risk of bias (43). Their methodological concerns included poor data availability on randomization and blinding process, deviations from the intended intervention, potentially missing outcome data, and some discrepancies in reporting results. Notably, most of the papers presented the results according to the per-protocol analysis (31–36, 38, 41, 43), which might have led to the increased bias. The attempt was made to perform the quantitative synthesis of the short-term effectiveness of corticosteroids in the first episode of bronchiolitis compared between RSV-positive and RSV-negative groups; however, we encountered a significant heterogeneity that heterogeneity (I^2^>75%) that any potential subgroup analysis could not have avoided.

### Population

The age of eligible participants differed between the included studies. Three studies, all based in the USA, recruited infants up to 12 months of age (31, 39, 40). The remaining studies included children up to 15 (34), 18 (32, 35) or 24 (33, 36, 37, 41, 43) months of age. One of the RCTs initially analyzed children under the age of 3 years; however, according to predefined inclusion criteria, we decided to selectively assess only the patients under the age of 2 years (38). All studies included patients with wheezing as one of the compulsory criteria apart from one (32), in which crackles or wheezing were needed on inspection. Six studies were conducted in the ED units and the pediatrics wards (35–37, 39–41), while six of the studies recruited solely hospitalized children (31–34, 38, 43).

### Viral detection

Most of the identified studies performed viral detection tests only on the presence of RSV (31, 32, 34–37, 39, 40). The other additionally detected viruses included HRV, bocavirus (33, 38), influenza virus (33, 38, 41, 43), parainfluenza virus (33, 38, 43), and adenovirus (33, 38, 41, 43). Moreover, the viral detection techniques varied across the studies, i.e., virus isolation, serology testing, rapid immunoassay method of nasopharyngeal swabs, and PCR tests. Two out of twelve studies did not provide the results with an adjustment to the performed viral testing (34, 35).

### Interventions

The systemic corticosteroids were administered orally in all the included studies, apart from one in which intramuscular injections were used (31) (**Table 1**). Prednisolone served as the intervention drug in five studies (33, 35, 36, 38, 43), while dexamethasone was used in the remaining seven (31, 32, 34, 37, 39–41). Usually, prednisolone was given orally at 2 mg/kg/day (33, 35, 36, 38), while in one study decreasing dosages starting from 15 mg to 2,5 mg were administered (43). Furthermore, the heterogeneity across the duration of the interventions was noticed. A single dose of corticosteroids was administered in three studies (37, 39, 41). The 3-day intervention course was introduced in five RCTs (31, 33–35, 38). The 5-, 6-, or 7-day treatment with corticosteroids was tested in four studies (32, 36, 40, 43).

### Short-term outcomes of corticosteroid treatment in severe bronchiolitis per viral etiology

Only two studies showed significant differences in the short-term efficacy of systemic corticosteroids compared between RSV-positive and RSV-negative groups (n=313) (32, 38).

In one of these studies, the mean duration of hospitalization of neither RSV-positive nor HRV-positive patients administered corticosteroids varied from the ones receiving a placebo (mean ± SD [hours]: 57,2 ± 39,9 vs. 47,6 ± 38,7 for RSV-positive and 14,6 ± 32,5 vs. 19,5 ± 31,9 for HRV-positive, both P>.05) (38). However, in the RSV-negative group, oral prednisolone almost by one day shortened the mean length of hospital stay compared to the placebo group (mean ± SD [hours]: 19,7 ± 31,5 vs. 40,0 ± 31,3, P=.002). Furthermore, the mean hospitalization time significantly differed between RSV-positive and HRV-positive children in the intervention group (mean ± SD [hours]: 57,2 ± 39,9 vs. 19,7 ± 31,5, P=.002) (38).

In the second study, oral dexamethasone’s efficacy, regarded as the readiness rate for discharge at 48 hours, was higher in the RSV-negative group than in the RSV-positive group (OR 0.28, CI95% 0.09-0.92, P=.03) (32).

Four studies reported no difference in the hospital admission rate after the initial visit to ED between RSV-positive and RSV-negative children who were administered systemic corticosteroids (37, 39–41).

### Chronic outcomes of corticosteroid treatment in severe bronchiolitis per viral etiology

The long-term follow-up – herein defined as two-month or more extended time – was evaluated only in three studies (33, 35, 38). The prevalence of respiratory symptoms during the two-year follow-up by Berger et al. (35) was not reported according to the RSV infection status. Solely Jartti et al. reassessed their participants two (38) and 12 months (33) after their studies’ enrollment. Although in their report, the number of wheezing episodes and initiation of regular controller medication for asthma symptoms within 12 months was not significantly reduced in the HRV-positive intervention group, in HRV(+) children less risk of physician-confirmed recurrence within 2 and 12 months was shown in the prednisolone group compared with placebo (both P<.05) (33). Moreover, they observed fewer new wheezing episodes during the 12-month follow-up in the corticosteroid-treated group compared with the placebo (P=.04) (33).

## Discussion

Although this systematic review failed to provide evidence in favor of the use of systemic corticosteroids in children with severe bronchiolitis, it has identified a trend of the positive association between the use of systematic corticosteroids and duration of hospitalization in RSV-negative infants in hospitalized bronchiolitis (32, 38).

The scarcity of expected effect may be attributed: (i) to the considerable diversity in methodology among the analyzed studies, (ii) under-reporting the full panel of respiratory viruses in study subjects, (iii) baseline inflammatory endotype of the child rather than only the viral agent, and (iv) that most studies focused on RSV etiology. Only two studies from the same study center reported the details on HRV detection – the second most common cause of bronchiolitis in infants above six months old. These studies showed oral prednisolone as an effective modality in managing the first HRV-induced wheezing episode. This effectiveness was defined as clinical improvement (no escalation to non-invasive ventilation) and reduced relapses during the first months of follow-up (33, 38). Therefore, our analysis highlights the lack of predictive enrichment strategies in trials investigating corticosteroid treatment in bronchiolitis, which is in line with a recent meta-analysis by Elliott et al. (6).

On the other hand, the strengths of this study are in the identification of the existing evidence gaps, in the innovative view on the corticosteroid’s treatment in bronchiolitis, including focusing on both the first episode and its viral etiology, and efforts made to contact the authors to address the missing data. Also, there were attempts to synthesize the results quantitatively with subgroup analyses; however, the significant heterogeneity could not have been omitted.

We attempted to make the results of this systematic review as clinically relevant as possible. Hence, we dichotomized the data into RSV and non-RSV (another respiratory virus) data to accommodate the current clinical practice protocols that do not include mandatory testing for all respiratory viruses. Moreover, many confounding factors across the studies undermined the synthesis of the available data. The performed viral testing was obsolete, including viral isolation and serology tests (43). In some studies, most patients were RSV-positive (34, 43), or a noticeable proportion of participants did not undergo viral detection. A considerable loss to follow-up was found to be another confounder (35, 36, 41). Regarding the two Finnish studies, there was a delay in initiating the study’s drug administration due to the completion of HRV detection in the second study (33) compared to the first one (38) (45 vs. 0 h, respectively).

RSV remains the leading cause of severe bronchiolitis and, proportion-wise is the most prominent risk factor for future asthma development (42). The possible lack of action of systemic corticosteroids in RSV bronchiolitis may result from at least two reasons: firstly, several studies have shown that RSV can inhibit the immunosuppressive activity of corticosteroids *via* the glucocorticoid receptor (44). Secondly, dexamethasone was shown to have a favorable inhibitory impact on RSV-driven mucus production yet prevent immune responses that limit RSV infection *in vitro* and *in vivo* (45). On the other hand, one may expect a beneficial effect of systemic corticosteroid therapy in HRV-induced bronchiolitis due to the well-known Th2-skewing, a typical feature for atopic children infected with the HRV-C virus (46). Also, over the last decade, several cohort studies have associated an early life HRV infection with recurrent wheeze and asthma in the following years (11, 47–49).

Recent evolution in high-throughput sequencing technology offers an opportunity for personalized guidance in pharmacological management and assessing long-term respiratory sequelae in infants with bronchiolitis (11, 50, 51). A relatively small number of epidemiologic studies have investigated associations between biological (e.g., viral etiology, proteins), genetic and environmental factors in infants with bronchiolitis. Even fewer intervention studies in bronchiolitis incorporated a predictive enrichment approach using an interplay of such factors in their methodology.

In 2020-2021, we witnessed an unprecedented revolution in respiratory virus testing on a mass scale. A wide distribution of the mass PCR testing for severe acute respiratory syndrome coronavirus 2 (SARS-CoV-2) infections has contributed to the increased use of the multiplex technique for other respiratory viruses (52). Therefore one cannot exclude the notion that in the coming years, studies evaluating a panel of respiratory viruses will provide more evidence around the role of HRV as a potential biomarker for the use of selected medications, including corticosteroids, during the first episode of bronchiolitis (53).

According to our recent meta-analysis on the association between infant bronchiolitis and recurrent wheeze, it has been shown that HRV-bronchiolitis children were more likely to develop recurrent wheeze (OR 4.11) and asthma (OR 2.72) than RSV-bronchiolitis group (P <0.01) (54). Furthermore, it has been proposed that the impact of rhinoviral infection may be species-specific. Of three species (A, B, and C), HRV-C-induced bronchiolitis has been linked to the highest risk for preschool wheeze and asthma in children (10). Thus, future studies evaluating the treatment efficacy according to virus subtypes are eagerly anticipated.

We are aware of several limitations of our review. The heterogeneity of the severity of assessed bronchiolitis episode introduced interventions, pre-specified outcomes, and follow-up time of analyzed studies makes our results less precise. Even though the search strategy was inclusive, within the initially identified studies recruiting only first wheezing bronchiolitis, viral tests were rarely performed and mainly focused on RSV etiology. Hence, the number of finally included studies was sparse.

Severe bronchiolitis remains a heterogeneous disease ideally suited for a precision medicine approach. This systematic review shows that there is currently insufficient data to recommend using systemic corticosteroids for short-term beneficial effects in treating the first episode of bronchiolitis. Nevertheless, an identified trend of the positive association between the use of systematic corticosteroids and shorter duration of hospitalization in RSV-negative infants hospitalized with the first episode of bronchiolitis should be further elucidated.

In conclusion, our study points out the need to identify groups of infants who would benefit from systemic corticosteroid treatment. A precise definition of the group of patients who benefit from a treatment is associated with decreased use of non-specific treatment. Therefore, this approach could ultimately result in decreased overall usage of systemic corticosteroids in bronchiolitis. Predictive enrichment approaches are guided by integration multi-omic data on HRV infection status (with its subtyping). Ongoing research in this area should focus on elucidating the complex interactions between pathogenetic factors in viral bronchiolitis with the aim to increase effectiveness and prevent the development of chronic outcomes.

## Data availability statement

The original contributions presented in the study are included in the article/supplementary material. Further inquiries can be directed to the corresponding author.

## Author contributions

DA, HM, WF, TJ, and AW contributed to the study design. DA and HM performed the systematic search, analyzed the data, and wrote the manuscript. WF, AW, NP, TJ reviewed the first draft of the manuscript, gave directions around the table and the figure design, and worked on the editing of manuscript to help reach the final version. All authors contributed to the article and approved the submitted version.

## Funding

The Sigrid Juselius Foundation, Helsinki, Finland (D. Ambrożej and T. Jartti).

## Conflict of interest

The authors declare that the research was conducted in the absence of any commercial or financial relationships that could be construed as a potential conflict of interest.

## Publisher’s note

All claims expressed in this article are solely those of the authors and do not necessarily represent those of their affiliated organizations, or those of the publisher, the editors and the reviewers. Any product that may be evaluated in this article, or claim that may be made by its manufacturer, is not guaranteed or endorsed by the publisher.
